# Principal component analysis for designed experiments

**DOI:** 10.1186/1471-2105-16-S18-S7

**Published:** 2015-12-09

**Authors:** Tomokazu Konishi

**Affiliations:** 1 Department of Bioresource Sciences, Akita Prefectural University, Shimoshinjyo Nakano, Akita 010-0195, Japan

**Keywords:** experimental data, centering, generality, principal component analysis, training data, multivariate analysis

## Abstract

**Background:**

Principal component analysis is used to summarize matrix data, such as found in transcriptome, proteome or metabolome and medical examinations, into fewer dimensions by fitting the matrix to orthogonal axes. Although this methodology is frequently used in multivariate analyses, it has disadvantages when applied to experimental data. First, the identified principal components have poor generality; since the size and directions of the components are dependent on the particular data set, the components are valid only within the data set. Second, the method is sensitive to experimental noise and bias between sample groups. It cannot reflect the experimental design that is planned to manage the noise and bias; rather, it estimates the same weight and independence to all the samples in the matrix. Third, the resulting components are often difficult to interpret. To address these issues, several options were introduced to the methodology. First, the principal axes were identified using training data sets and shared across experiments. These training data reflect the design of experiments, and their preparation allows noise to be reduced and group bias to be removed. Second, the center of the rotation was determined in accordance with the experimental design. Third, the resulting components were scaled to unify their size unit.

**Results:**

The effects of these options were observed in microarray experiments, and showed an improvement in the separation of groups and robustness to noise. The range of scaled scores was unaffected by the number of items. Additionally, unknown samples were appropriately classified using pre-arranged axes. Furthermore, these axes well reflected the characteristics of groups in the experiments. As was observed, the scaling of the components and sharing of axes enabled comparisons of the components beyond experiments. The use of training data reduced the effects of noise and bias in the data, facilitating the physical interpretation of the principal axes.

**Conclusions:**

Together, these introduced options result in improved generality and objectivity of the analytical results. The methodology has thus become more like a set of multiple regression analyses that find independent models that specify each of the axes.

## Background

Principal component analysis is frequently used to transform matrix data consisting of multiple measured items. To represent the data efficiently, the first principal axis is fitted to the matrix, minimizing the distances between the data and the axis; then the next axis perpendicular to the first axis is fitted to the data, and so on [1-3]. Since there may be interdependence between the measured items, a limited number of orthogonal axes will represent much of the original data; thus, the transformed matrix can summarize the original data with fewer dimensions. Additionally, the summarization process is objective and has the least number of options in the calculation. For clarity, it is possible to describe this process by the singular value decomposition of the matrix [2]. Let **X **be a sample-by-item matrix, containing items with zero mean. The matrix can be factorized into one diagonal and two unitary matrices, **X **= UDV^*^. The unitary matrices **U **and **V **control the directions of the principal axes, while the diagonal matrix **D **records the singular values. The principal components for the samples in **X **are defined as **Y**_s _= **XV **(= **UD**). The unitary matrix **V **is frequently used as loadings for the components; however, the principal components for the items, **Y**_i _= X^*^U (=**VD**), will be used here instead.

As discussed below, the principal component methodology is unsuitable for the analysis of experimental or diagnostic data. One reason is that the method is sensitive to noise and bias in the samples; the method is intrinsically weaker with noise compared to factor analysis, which contains error terms. Additionally, because of how distance is defined [1], both singular value decomposition and principal component analysis are sensitive to outliers. Various robust alternatives have been produced [2-4] and they are applied to microarray data [5]. However, sensitivity to noise and to outliers could be separate issues, since the noise of concern here is due to small individual differences among samples in many items, not due to outliers in particular items. Despite this weakness, principal component methodology cannot take advantage of an experimental design planned to reduce the effects of noise. Indeed, it assumes an equal weight for all samples in **X**, even though these samples may have different weights and dependencies. Hence, noise tends to affect the resulting components. Skinner et al. (1986) identified this instability in the directions of the axes in the context of sampling design; since experimental designs often consist of repeated measurements, the effects of the weights and independencies among the data should become larger [6]. Konishi and Rao (1992) introduced a superior data handling methodology that contains families with differing numbers of siblings [7]. However, the design of experiments could be biased in the families as well; for example, some experiments and examinations in toxicology or biopsy could include huge numbers of samples for a particular group of chemicals or clinical conditions. The resulting bias would affect both the directions of the principal axes and the unit of size, as will be observed in the examples presented below.

Another disadvantage is with the method's generality; the identified principal components are only valid for the given set of data. A comparison or integration of principal components requires specialized assumptions, for example, that the experiments or observations to be integrated share common data, and that the experiments differ only in their means [2,3]. Indeed, Alter et al. (2003) applied the method to a pair of experiments that shared a common set of samples [8]. However, these assumptions are only suitable for specialized combinations. Additionally, the physical interpretation of the axes in the context of an experiment is not obvious in many cases. Therefore, comparing or even approximating the components for different experiments is difficult. The lack of generality for principal components conflicts with the goal of most experiments, which are often planned to examine hypotheses and discover theories with generality. This also is problematic for the natural sciences, which requires integration of knowledge obtained from various investigations. Furthermore, generality is expected in diagnosis or toxicology studies.

Here, modifications to the method are introduced which allow generality to be achieved in the results. First, the processes of identification and application of the axes were separated, enabling the axes to be shared among experiments. The unitary matrices that assign the axes are discovered using training data, which consist of representatives of the groups in the experimental design. A representative is found as the mean of a group, such as repeated measurements of a treatment. By taking means of repeats, the level of measurement error is reduced. By using robust methods such as the trimmed mean or median, the effect of outliers can be further reduced. In addition, by assembling sets of data into appropriate groups, bias due to unbalanced data among the groups can be removed. Second, the center of the data **X **was determined in accordance with the experimental design. The *de facto *standard of the center is the sample means, i.e., the item-wise means of all the samples; however, these means are not always the best choice because they are sensitive to the biases in the groups. Rather, many experimental designs have a control group that is suitable as the center. The introduced options for the training data and the center are used to improve the fidelity of the methodology to the design of experiments. With these revisions, the components present differences of the samples from the control group, but not the data fluctuations estimated by the standard deviation of data. Third, the effects of the size of **X **on the components were eliminated. Two categories of principal components, i.e., that of samples and items, are used to present the data, instead of the original biplot. Both categories are closely related, because one is the source of the other, and they can be represented on identical axes. Taken together, each axis would assign an independent multiple regression model. On an axis, differences from the control group are presented from a unique perspective.

The effects of these modifications were observed in two microarray experiments. Since the microarray measures a very large number of items, e.g., the expression level of genes, methods to summarize the data are beneficial [9]. Indeed, principal component analysis has been used for an exploratory estimation of the underling structure of microarray data [10,11]. In particular for pattern recognition and clustering, information for the data magnitude, **D**, is not necessarily required; hence, the unitary matrices could be used instead of the principal components [12]. Nevertheless, defects in the original methodology limit the application to microarray data regardless of whether **D **is used, because microarrays are often used for experiments or examinations in which a set of data contains groups of several repeated measurements, and these groups are frequently biased. As microarrays are often used in large projects, the resulting knowledge should be shared widely and integrated seamlessly. Here, the effects of the introduced options are observed in the separations of the groups and their robustness to noise. Additionally, the appropriateness of the cancellation of the size effect becomes obvious by the constancy in the range of the components presented. There also exists the possibility of sharing axes, which enables the use of a common framework among experiments.

## Methods

### Preparation and pretreatment of the data matrix

This work used experiments that investigated several groups using repeated measurements. The data appeared in the Gene Expression Omnibus [13]: a time course in mammary gland development, GSE8191 that was carried out by Anderson et al. (2007) [14], and expression data from a rat liver 48 hours after treatment with different toxic compounds, GSE5509 that was carried out by Spicker et al. (2008) [15]. Perfect match sample data were parametrically normalized, and gene expression levels were estimated by summarizing the normalized data by taking the trimmed mean as was described in my previous study [16]. The process was performed using the SuperNORM data service (Skylight Biotech Inc., Akita), and the normalized perfect match data and summarized gene data are available in Gene Expression Omnibus under accession of GSE31375. The significance in the expressional changes was tested using a two-way analysis of variance on the normalized perfect match data, assuming a linear relationship: (data difference) = (probe sensitivity) + (group effect). A threshold of 0.01 was used for the two-sided test.

### Settle the reference of experiment as the center of data

The matrix of expression levels is subtracted using the reference data,

(1) $$X=\left(\begin{array}{ccc} s_{11} & \cdots \; & s_{1m}\\ \vdots & \ddots & \vdots \\ s_{n1} & \cdots \; & s_{nm} \end{array}\right)-\left(\begin{array}{ccc} r_{1} & \cdots \; & r_{m}\\ \vdots & \ddots & \vdots \\ r_{1} & \cdots \; & r_{m} \end{array}\right)$$

where *s *is the normalized sample data, *r *is the reference found for each item, and *n *and *m *are the number of samples and items, respectively. The reference, which determines the origin of the principal components, were found as the item-wise means of all the samples for the time-course data, or of the control group for the toxicology data. Missing data in **X **were replaced with zero; the same replacement was performed to remove certain items, such as negative genes in the analysis of variance test. This replacement is based on a fail-safe design: as zero elements in **X **indicates that there is no difference from the reference, the replacement of certain values with zero tends to move the resulting principal components closer to the origin, but not distally, which may suggest positive results.

### Scaling of principal components

In **X**, the number of functional items, *m*_f_, could differ among samples due to missing data; hence, differences in *m*_f _should not affect the size of the principal components so that comparability is maintained. Such differences in *m*_f _would be much larger when comparing results obtained from different sets of items, for example, whether test-negative genes are included or not. However, the size or magnitude of the principal components depends on *m*_f_, as follows.

Theorem 1. Let *y *be an element of the principal components for samples, **Y**_s_, of a matrix **X **that contains *m*_f _functional items, and *v *be an element of the unitary matrix **V **derived from **X**. For the *j *th component of the *k *th sample, *y_kj _*∝ *m*_f_^1/2^.

Proof. Since **V **is a unitary matrix, V^*^V = **I**, thus $\overset{m_{f}}{\underset{l=1}{\sum }}{\left(v_{lj}\right)}^{2}=1$ for any *j*. Hence, *v_j _*∝*m*_f _^-1/2^. According to the definition of principal components, the value of *y_k _*for the *j *th component is calculated as $y_{kj}=\overset{m_{f}}{\underset{l=1}{\sum }}x_{kl}v_{lj}$. Hence, $y_{kj}\propto m_{f}{\bar{x}}_{k}\times m_{f}^{-1/2}={m_{f}}^{1/2}{\bar{x}}_{k}$, where ${\bar{x}}_{k}$ is the average of *x_k_*. We can assume stability of ${\bar{x}}_{k}$ regardless of the selection of items, since ${\bar{x}}_{k}$ is estimated from centered data (1). Therefore, $y_{kj}$ is proportional to ${m_{f}}^{1/2}$. □

To maintain the principal components' compatibility regardless of the size of *m*_f_, the components should be scaled. After a singular value decomposition is performed on the matrix with *m*_f _functional items, the scaled components for the samples are estimated as

(2) $${\text{Z}}_{\text{s}}={m_{\text{f}}}^{-1/2}{\text{Y}}_{\text{s}};$$

according to Theorem 1, this will unify the magnitude of PC. Although scaling in principal component analysis tends to be employed in the context of the pre-scaling of data or multidimensional scaling for biplots [2,3], the method proposed here finds the average contribution of each element to the principal components. As this scaling will not cancel the singular values of **D**, the ratios among the axes will be retained. In the same manner, the ratios for the items are scaled as **Z**_i _= *n*_t_^-1/2^**Y**_i_, where *n*_t _is the number of data samples of the matrix in which **U **was found.

### Locating orthogonal axes in training data

The axes were identified in a set of predetermined training data matrices that were intentionally chosen. Unless otherwise stated, the training data **T **was the set of the sample means from each group, for example, each point in the time-course data or each chemical in the toxicology data, subtracted with the reference as in (1). The axes were found using singular value decomposition, **T **= **U**_T_**D**_T_**V**_T_^*^. The principal components for the samples are defined as **Y**_s _= **XV**_T_, and those for the items are defined as **Y**_i _= T^*^U_T_. Additionally, the effects of robust alternatives to singular value decomposition algorithms were observed using two different functions in R (R development Core Team, 2011): [17] the *robustSvd *function (Liu, et al., 2003) [5] in the *pcaMethods *library [18] and the *PcaHubert *function (Hubert, et al., 2005) in the *rrcov *library [19]. In the data analysis examples presented here, the directions of the corresponding axes were unified within the decomposition results by manually reversing the signs of some of the columns of **Y**_s _or **Y**_i_. The obtained components were scaled as in the previous section. The source code for R is available as Additional file 1.

## Results

### Improvement in separating repeating groups

**Figure 1:**
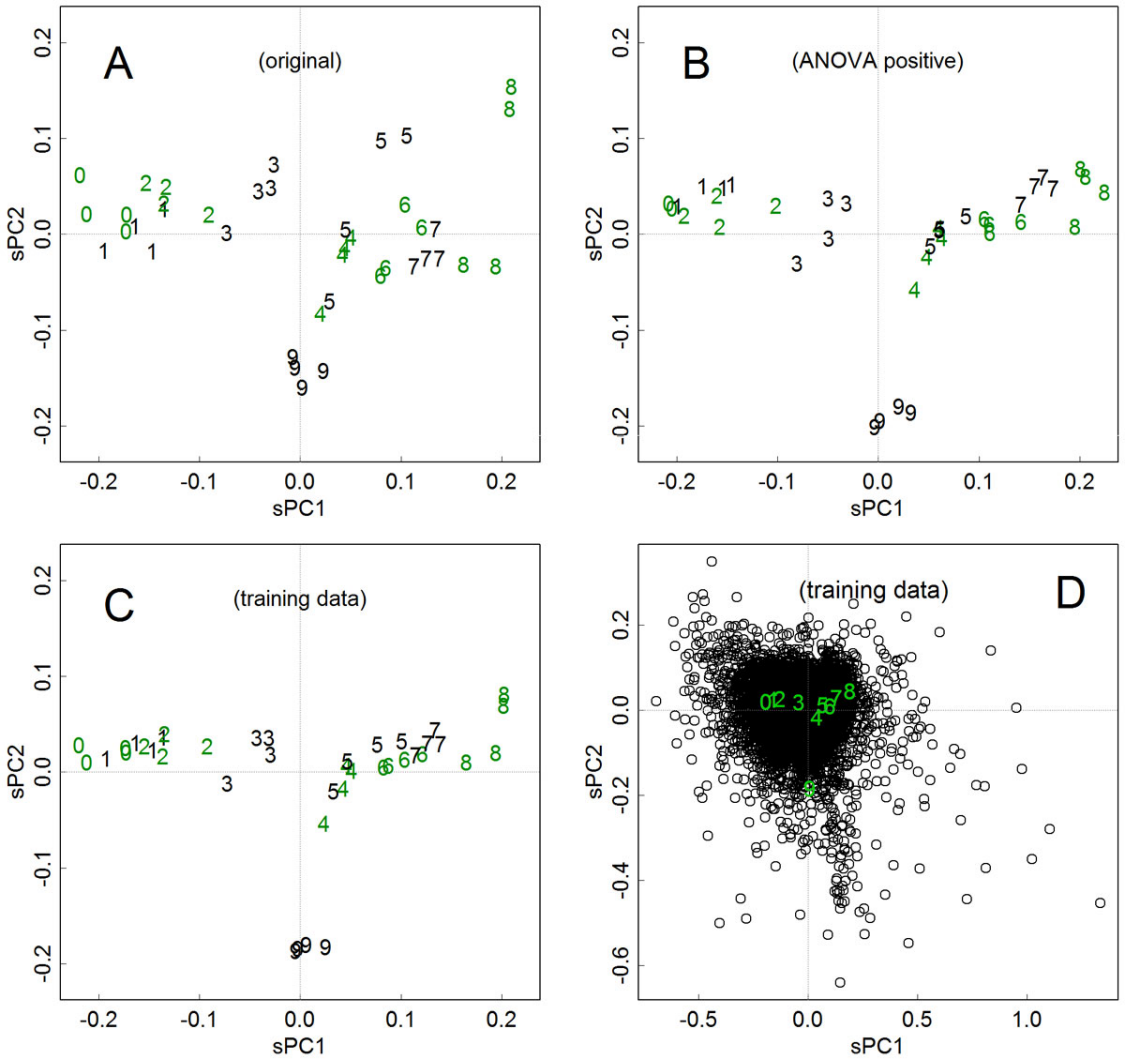
**Scaled principal components for samples in the time-course experiment, with the axes found in different sets of data**. Data were obtained for mammary gland development (Anderson et al., 2007); groups 0 to 5: days 1 to 19 of pregnancy; groups 6 to 8: days 1 to 9 of lactation, group 9: day 2 of involution [14]. **A**. Results of the original method. The axes were found with all samples of 12,487 genes. Scaled principal components (sPC) of the first and second axes are shown. **B**. Axes were found in 5,892 positive genes with the analysis of variance test. **C**. Axes were found in the training data, prepared by using each group's sample means of 12,487 genes. D. An example of biplot-like presentation. Scaled principal components for the training data for gene items (open circles) and groups (0 to 9) are presented in a same scale.

The effects of using training data and the focus on the positive genes of the test were investigated in mammary gland development data taken from Anderson et al. (2007), with a view towards group separation [14]. In the time-course experiment, ten groups of time points were measured: pregnancy (groups 0 to 5), lactation (groups 6 to 8), and involution (group 9). The scaled principal components for samples on the first and second axes are presented in Figure 1. Compared with the original method that found axes for the full data matrix **X **(Figure 1A), the separation of groups was improved when axes were found for selected genes (Figure 1B) or for the training data (Figure 1C). The samples for each group were located nearer to each other, creating better separation between the groups; this improvement is especially obvious in the second axes. Accordingly, the axes are self-explanatory in Figures 1B and 1C; the developmental stages of the mammary gland appeared in a straight line along the first axis, and the involution appeared separately along the second axes. Also, while the axes were calculated with only half of the genes in Figure 1B, the range of the components remained the same. This demonstrated the appropriateness of scaling the components.

The alternative algorithms, which are robust to outliers, did not improve the group separations (Additional file 2). In the two cases, using all the genes and using only the ANOVA-positive genes, the separation of groups degraded. This inefficiency demonstrates that robust algorithms cannot solve the problems in the application to experimental data. This could be due to individual differences, which primarily cause data noise, because they may not appear as outliers in particular genes but only as the smaller differences in many genes [16]. Additionally, by pre-scaling **X**, the group separation degrades (Additional file 2). This suggests that in microarray data, pre-scaling would enhance the effects of noise.

### Robustness to individual differences among samples

**Figure 2:**
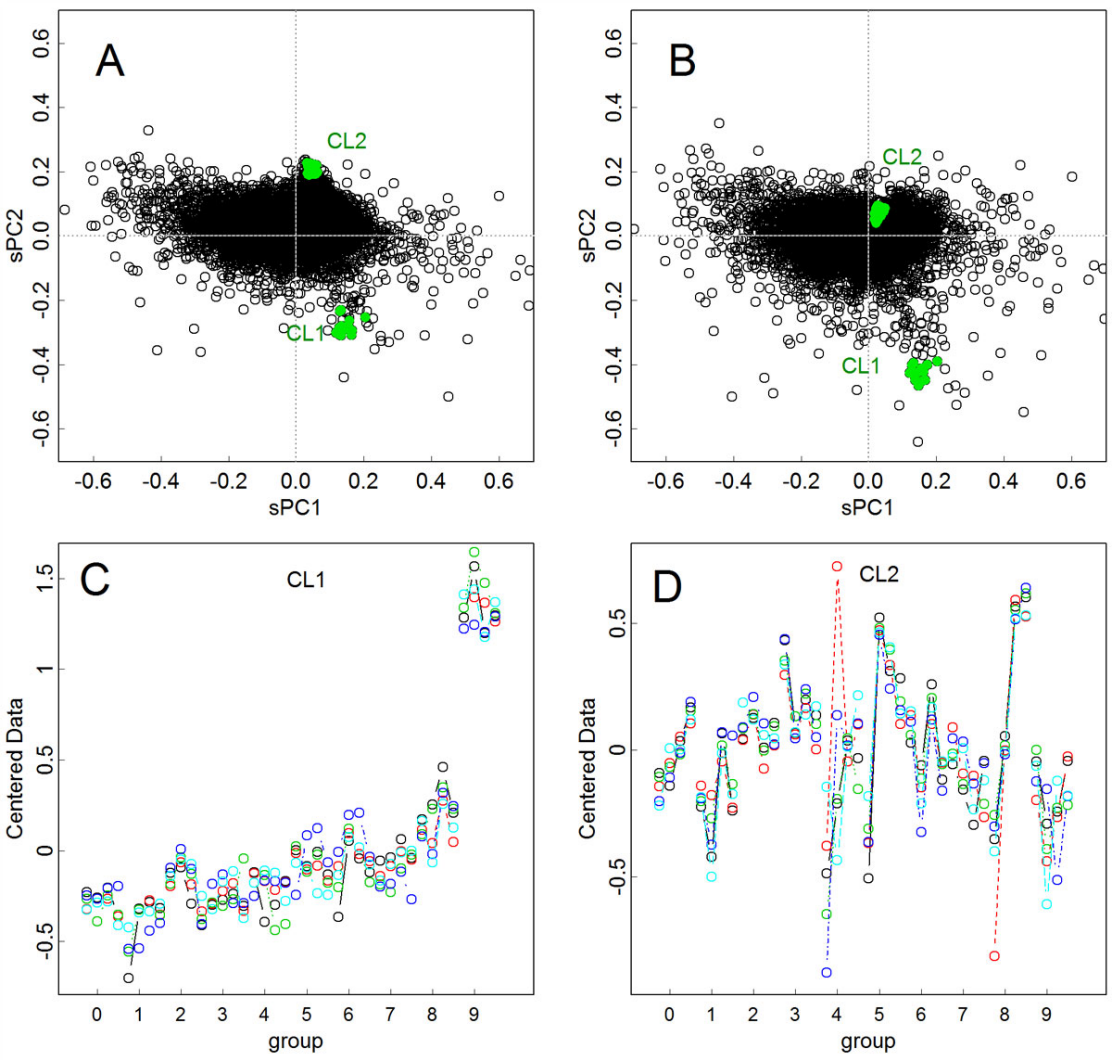
**Effect of training data on the scaled principal components for items**. **A**. Results of the original method, corresponding to Figure 1A, showing the two clusters, CL1 and CL2. **B**. Axes were found in the training data, corresponding to Figure 1C. **C**. Expression levels of five genes randomly selected from CL1. **D**. Those selected from CL2, showing higher within group variances.

The effects of using training data were further investigated on the principal components for the items. Figures 2A and 2B show the components found in the original matrix and in the training data, respectively. Let us focus on two clusters of genes, CL1 and CL2; these genes are located in both of the graphs at slightly different positions. In Figure 2A, both clusters were outside of the large cluster at the origin of the graphs, which was the gathering of genes that did not show large expressional changes. In contrast, with the axes found in the training data, cluster CL2 was located inside of the center cluster (Figure 2B). Five genes were randomly selected in both clusters, and their expressional changes are presented in Figures 2C and 2D. In cluster CL1, the expression levels changed rather uniformly, while the genes in cluster CL2 showed larger differences between the samples. Since the effect of the outlying samples have been reduced by taking sample means in the training data, the genes in cluster CL2 were located closer to the origin in Figure 2B, showing robustness to the outlying samples.

### Robustness to biases in the groups

**Figure 3:**
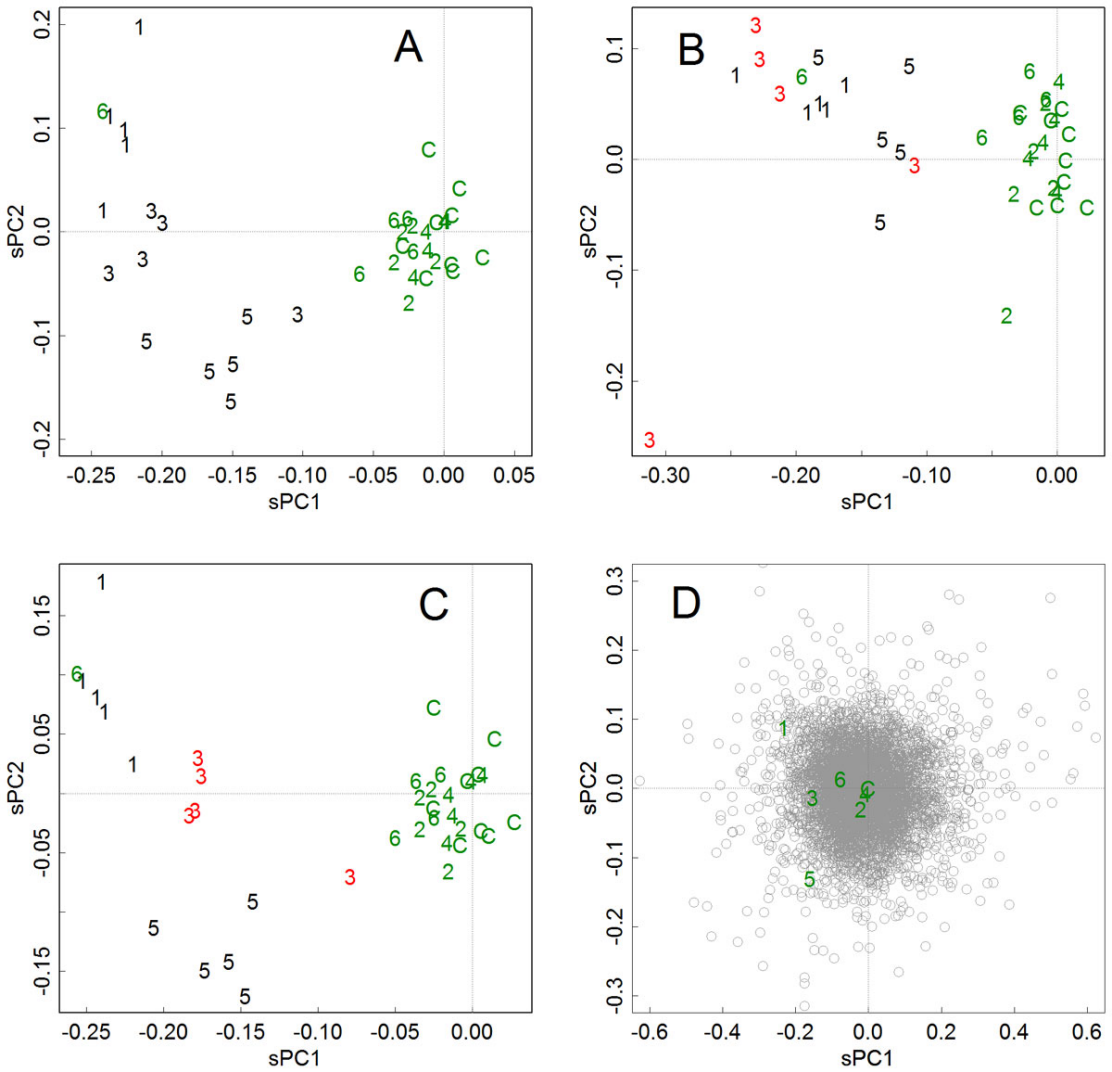
**Scaled principal components for samples in toxicology data measured by Spicker et al**. (2008), found in various training data sets [15]. Groups 1, 3, and 5 were those for carcinogens, groups 2, 4, 6 were those for nontoxic compounds, and C was the mock control. **A**. Training data was prepared by using all of the groups' sample means. **B**. To mimic bias, the sample means of group 3 were replaced with all the samples. The increased sensitivity towards the group 3 samples is obvious, especially in the second axis. **C**. Training data were prepared without group 3, and the data of group 3 were subjected as unknown samples. The carcinogens including group 3 were separated from the nontoxic groups. **D**. An example of biplot-like presentation. Scaled principal components of the training data for genes and groups are presented in a same scale.

The effects of training were further studied using toxicology data. Spicker et al. (2008) measured three toxic chemicals (groups 1, 3, and 5), three nontoxic chemicals (groups 2, 4, 6), and one nontoxic control; there were five repeats [15]. Besides the training data set that included all of the chemicals' sample means, two alternative sets of training data were prepared. One was used to simulate bias in groups. To accomplish this, in one of the toxic compounds (group 3) the sample means were replaced with all the samples; hence, the training data contained six group means and five group 3 samples. The calculations were performed using only the positive genes in the test. In the axes of the original training data, the first axis separated the toxic and nontoxic chemicals, and the second axis separated the toxic chemicals (Figure 3A). However, in the artificially-biased training data, the first axis emphasized group 3 with a higher magnitude of the principal components, and the second axis separated the group 3 samples (Figure 3B). It is obvious that the bias had unnecessarily increased the importance of group 3, by emphasizing rather trivial differences between the samples.

### Classifying unknown samples by applying previously found *V*_T_

Another training data set was used to investigate the application of previously found or even shared **V**_T_, for estimating the principal components for unknown samples. The training data was prepared using all sample means except those from group 3. The obtained first axis successfully separated the group 3 samples from the nontoxic chemicals, and the second axis separated the toxic chemicals (Figure 3C).

## Discussion

Enhancements to principal component analysis were introduced in this paper: separating the identification and application of the unitary matrix **V**, developing options in the training data and the reference, and scaling the components. The use of training data improved the resolution between groups (Figure 1). This improvement may have been derived by the reduction of noise effects; indeed, a similar improvement was observed by focusing on analysis of variance positive genes (Figure 1B); de Haan et al. (2007) have reported the same noise reduction effects [20]. Additionally, robustness was also achieved in the identification of groups of genes using principal components for the items (Figure 2). The superiority of using training data is seen in the robustness to sample biases. These bias effects are obvious in Figure 3B, and the problem cannot be solved by focusing on certain genes; rather, it can be simply avoided by selecting appropriate groups for the training data (Figures 3A and 3C).

The introduced options may somewhat reduce the objectivity of the original method; however, the total objectivity of the methodology may be preserved. In the original method, both **U **and **V **and hence **Y**_s _and **Y**_i _are calculated rather automatically for the given **X**. On the other hand, preparing a training data set and the reference requires a selection of samples and/or groups, which may reflect the analyst's ideas. However, each experiment has its own design for the grouping of data. The first choice for the reference should be representative of the control group, and those for the training data should be representatives of the experimental groups. In addition, the appropriateness of the data and group selections can be verified by comparing the results for alternative selections; even if potential alternatives are not found, the falsifiability can be retained.

This comparable character of the scaled components is also beneficial for verification, since as expected, the scaled version shows likely ranges with those calculated with different numbers of items (Figures 1B and 1C). Actually, the use of proper training data resulted in a better separation of groups, facilitating interpretation of the axes (Figure 1). It should be noted that the original method is objective in the calculation processes, but not in interpreting the physical meaning of the axes. If the introduced options result in the axes becoming self-explanatory, the objectivity of the analysis would be improved in total.

The options improved the methodology by resulting in superior generality of the results. In the original method, a closed intelligent framework is formed in each set of data, since the resulting unit of size and directions of principal axes are valid only within the original data set. Therefore, the components and obtained knowledge are difficult to integrate among experiments. Here, the scaling of the components unified the unit of size. In addition, separating the identification and application of the axes enabled the same unitary matrices **V**_T_, which define the directions of the axes, to be shared between samples obtained in various experiments or laboratories. By sharing the axes and the unit size, laboratories can use a common framework. Indeed, the prearranged training data could classify unknown material appropriately (Figure 3C). Trivial differences between experiments could be cancelled using an appropriate reference found in each experiment. This type of usage is especially beneficial in applications for diagnosis and toxicology applications, enabling the classification of subjected samples. Also, we can compare responses found in particular conditions by swapping the unitary matrices between different studies. Such compatibility is advantageous for achieving deeper understanding of experimental data.

Another benefit to the scaling is that it allows biplot-like presentations. According to Jackson's definition, biplots present *c***UD **and (1-*c*)DV^*^, where 0<*c *<1; they are complementary and can reproduce information for **X **[2]. However, if we present principal components for the samples and items together, the information for the size fully appears in both of them; thus, differences in the magnitude of the axes will become quantitatively obvious. This is also beneficial to determining relationships between particular samples and items. For example, genes that actually separate group 9 of Figure 1C could be found from Figure 1D. Indeed, genes outlying in the second axis showed such an expression pattern (Figure 2C), and such relationships are easily found in plots like Figures 1D and 3D. Although the ranges of **Y**_s _and **Y**_i _could differ largely especially when the numbers of samples and items are distinctively different; however, those in the scaled **Z**_s _and **Z**_i _will become similar, hence they can be presented together.

By separating finding and applying the axes, the methodology has become analogous to a set of multiple regression analyses; each of the axes shows an independent regression model. On an axis, principal components for group representatives show which target is estimated. The principal component for a sample is a result of the regression; it shows the tendency of the sample to the estimated target. Components for the items show which items are contributive for the regression model. For example, PC1 for the mammary gland development data, which is assigned on the X-axis in Figure 1C, represents gland development; the value increased as pregnancy and lactation proceeded, and rapidly decreased after the termination of lactation. Many of the genes having the highest scores (located on the right side of Figure 1D) were those related to lipid synthesis, such as adjusting saturation of fatty acids, which are major components of milk. Coefficients of the regression model, stored in a column of **V**, multiply the gene items of a sample stored in a row of **X**, to find the target estimation of the regression function. The estimation of the component of a sample is stored in the corresponding column of **Y**_s _and **Z**_s_. The contributive gene item gets a high score in the corresponding columns of **V**, **Y**_i_, and **Z**_i_. However, this methodology is not purposive in finding out certain relationships of interest, unlike in multiple regression analysis. It represents relationships between groups rather objectively. Therefore, the analyst has to ascertain the proper axis by observing separation of the group scores.

## Competing interests

The authors declare that they have no competing interests.

## Supplementary Material

Additional file 1 **R scripts**. The complete scripts to import, analyze, and output the data. Click here for file

Additional file 2 **Results of principal components for samples, calculated by using other methods or conditions**. **E**. Results of the *robustSvd* function [5] to the whole set of 12,487 genes. Scaled principal components (sPC) of the first and second axes are shown. **F**. Axes were found in 5,892 positive genes with the analysis of variance test. **G**. Results of the *PcaHubert* function [19] to the whole set of 12,487 genes. **H**. Axes were found in the 5,892 positive genes. **I**. Effect of pre-scaling on the data. The whole set of 12,487 genes of centered data were divided by standard deviation before subjected to PCA. **J**. Axes were found in the 5,892 positive genes. Click here for file

## Declaration

The publication costs for this article have been funded by the Faculty of Bioresource Sciences, Akita Prefectural University, Japan.

This article has been published as part of *BMC Bioinformatics *Volume 16 Supplement 18, 2015: Joint 26th Genome Informatics Workshop and 14th International Conference on Bioinformatics: Bioinformatics. The full contents of the supplement are available online at http://www.biomedcentral.com/bmcbioinformatics/supplements/16/S18.
