# Supplementary material for: Principal component analysis for designed experiments
Source: BMC Bioinformatics. 2015 Dec 9;16(Suppl 18):S7. doi: 10.1186/1471-2105-16-S18-S7 (PMC4682404; doi:10.1186/1471-2105-16-S18-S7)
Supplement: Additional file 2 — Results of principal components for samples, calculated by using other methods or conditions. E. Results of the robustSvd function [5] to the whole set of 12,487 genes. Scaled principal components (sPC) of the first and second axes are shown. F. Axes were found in 5,892 positive genes with the analysis of variance test. G. Results of the PcaHubert function [19] to the whole set of 12,487 genes. H. Axes were found in the 5,892 positive genes. I. Effect of pre-scaling on the data. The whole set of 12,487 genes of centered data were divided by standard deviation before subjected to PCA. J. Axes were found in the 5,892 positive genes. [file 1471-2105-16-S18-S7-S2.pdf]

Figure S1: Results of principal components for samples, calculated by using other methods or conditions.

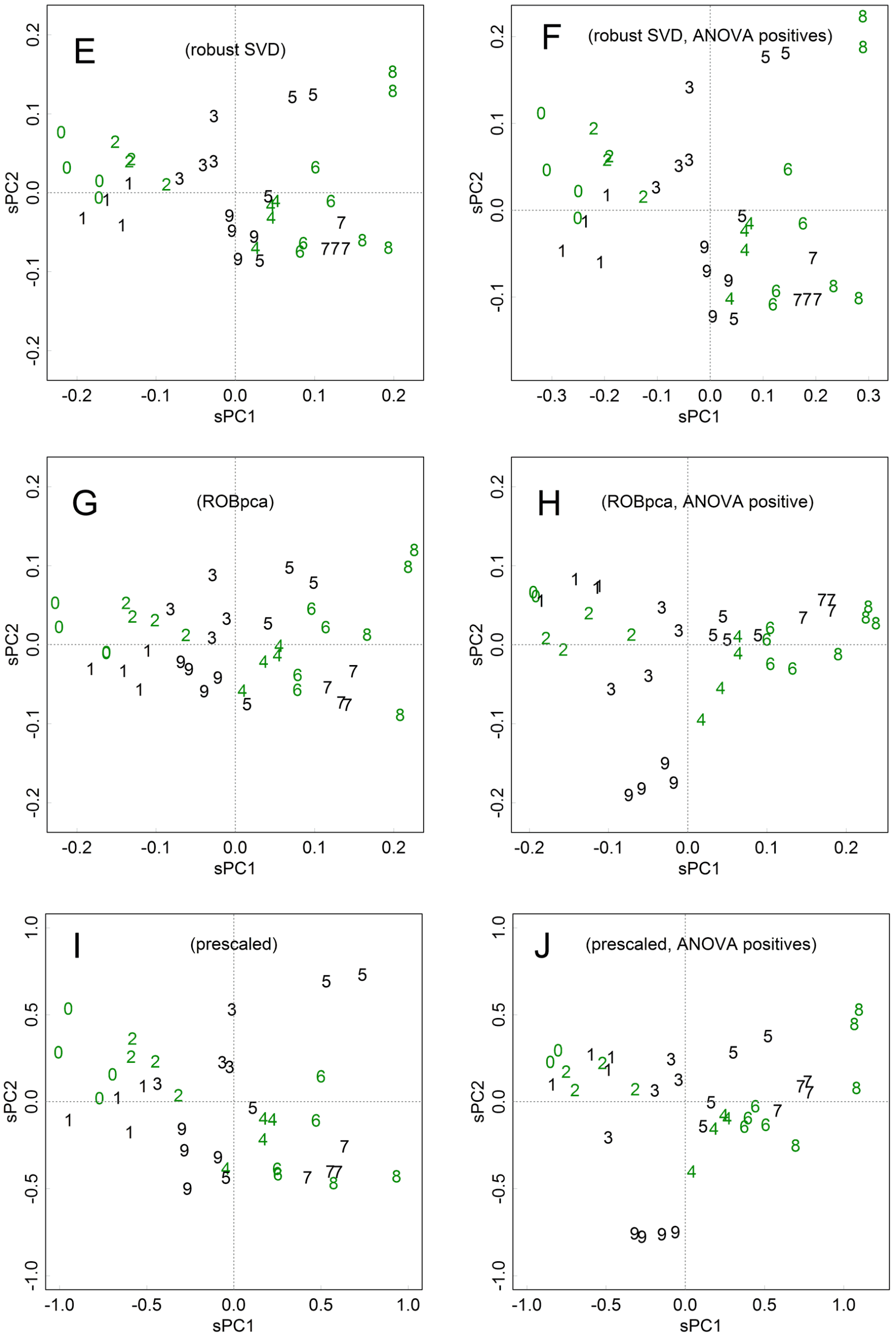

E. Results of the *robustSvd* function [5] to the whole set of 12,487 genes. Scaled principal components (sPC) of the first and second axes are shown. **F**. Axes were found in 5,892 positive genes with the analysis of variance test. **G**. Results of the *PcaHubert* function [19] to the whole set of 12,487 genes. **H**. Axes were found in the 5,892 positive genes. **I**. Effect of pre-scaling on the data. The whole set of 12,487 genes of centered data were divided by standard deviation before subjected to PCA. **J**. Axes were found in the 5,892 positive genes.
